# Role of radiotherapy in treatment of extramedullary relapse following total marrow and lymphoid irradiation in high-risk and/or relapsed/refractory acute leukemia

**DOI:** 10.3389/fonc.2022.1017355

**Published:** 2022-10-31

**Authors:** Colton Ladbury, Hemal Semwal, Daniel Hong, Dongyun Yang, Claire Hao, Chunhui Han, An Liu, Guido Marcucci, Joseph Rosenthal, Susanta Hui, Amandeep Salhotra, Haris Ali, Ryotaro Nakamura, Anthony Stein, Monzr Al Malki, Jeffrey Y. C. Wong, Savita Dandapani

**Affiliations:** ^1^ Department of Radiation Oncology, City of Hope National Medical Center, Duarte, CA, United States; ^2^ Department of Integrative Biology and Physiology, University of California Los Angeles, Los Angeles, CA, United States; ^3^ Department of Bioengineering, University of California, Los Angeles, Los Angeles, CA, United States; ^4^ Department of Physics, Emory University, Atlanta, GA, United States; ^5^ Division of Biostatistics, City of Hope National Medical Center, Duarte, CA, United States; ^6^ Department of Hematology and Hematopoietic Cell Transplantation, City of Hope National Medical Center, Duarte, CA, United States

**Keywords:** TMLI, ALL, AML, leukemia, radiation, relapse, salvage, HCT role of radiation following TMLI

## Abstract

**Background:**

Total Marrow and Lymphoid Irradiation (TMLI) is a promising component of the preparative regimen for hematopoietic cell transplantation in patients with high-risk acute myeloid leukemia (AML) and acute lymphoid leukemia (ALL). Extramedullary (EM) relapse after TMLI is comparable to TBI and non-TBI conditioning regimens. This study evaluates outcomes of patients treated with radiotherapy (RT) with EM relapse previously treated with TMLI.

**Methods:**

A retrospective analysis of five prospective TMLI trials was performed. TMLI targeted bones and major lymphoid tissues using image-guided tomotherapy, with total dose ranging from 12 to 20 Gy. EM recurrences were treated at the discretion of the hematologist and radiation oncologist using RT ± chemotherapy. Descriptive statistics and survival analysis were then performed on this cohort.

**Results:**

In total, 254 patients with refractory or relapsed AML or ALL were treated with TMLI at our institution. Twenty-one patients were identified as receiving at least one subsequent course of radiation. A total of 67 relapse sites (median=2 sites/patient, range=1-16) were treated. Eleven relapsed patients were initially treated with curative intent. Following the initial course of subsequent RT, 1-year, 3-year and 5-year estimates of OS were 47.6%, 32.7% and 16.3%, respectively. OS was significantly better in patients treated with curative intent, with median OS of 50.7 months vs 1.6 months (p<0.001). 1-year, 3-year and 5-year estimates of PFS were 23.8%, 14.3% and 14.3%, respectively. PFS was significantly better in patients treated with curative intent, with median PFS of 6.6 months vs 1.3 months (p<0.001). Following RT, 86.6% of the sites had durable local control.

**Conclusions:**

RT is an effective modality to treat EM relapse in patients with acute leukemia who relapse after HCT achieving high levels of local control. In patients with limited relapse amenable to curative intent, radiation confers favorable long-term survival. Radiation as salvage treatment for EM relapse after HCT warrants further evaluation.

## Introduction

Hematopoietic cell transplantation (HCT) is a form of consolidative therapy that is an essential component of potentially curative treatment regimens for patients with acute myeloid leukemia (AML) and acute lymphocytic leukemia (ALL). Much of the efficacy of HCT is attributed to conditioning with high-dose chemotherapy and, when possible, radiation therapy in the form of total body irradiation (TBI).

Given that the toxicity related to TBI is related to radiation dose to normal tissues, a more targeted form of TBI has been pioneered, called total marrow and lymphoid irradiation (TMLI), which aims to deliver radiation primarily to areas most at risk for leukemic involvement while being able to spare other normal tissues, thereby reducing toxicity (1). This has facilitated treatment of high-risk relapsed/refractory patients with active disease who otherwise would not have been candidates for transplant (2). Previous studies have shown that TMLI can permit target dose escalation while simultaneously limiting dose to critical structures, ultimately leading to more intense conditioning with less toxicity relative to TBI (3, 4). Organ sparing with TMLI has raised concerns of sparing of cancer cells and increased recurrence rates. We reported earlier on extramedullary recurrences in the first 101 patients with advanced refractory or relapsed acute leukemia undergoing allogeneic HCT with TMLI as part of the conditioning regimen at this center. This is a population of patients with more aggressive disease and higher tumor burden than patients undergoing traditional TBI. However, the risk of EM relapse using a TMLI-based conditioning regimen is comparable to that of standard TBI-based HCT conditioning regimens (5–9). Further, EM relapse does not appear to be dose-dependent. With a median follow-up of 12.8 months, 13 patients developed extramedullary relapses at 19 sites. Nine relapses occurred in the target region (≥ 12 Gy), 5 relapses in regions receiving 10.1 to 11.4 Gy and 5 relapses in regions receiving 3.6 to 9.1 Gy (9). Only EM disease prior to HCT predicted for EM relapse on multivariable analysis. Prior EM disease has also been found to be an independent risk factor in the setting of HCT with a TBI conditioning regimen, and therefore should not preclude these patients from undergoing TMLI regimen (6, 10–12). These data suggest the use of TMLI does not increase the risk of relapse in non-target regions.

Due to limited EM relapses in patients who have undergone TMLI-based conditioning, there is an additional question of how to treat relapses following conditioning with TMLI; though systemic therapy is a standard option (13, 14), select recurrences might achieve local control with additional radiation due to high response rates, which can prove to be either an effective salvage or palliative strategy (15, 16). In order to characterize the role of radiation in the treatment of extramedullary relapse, we performed a single-institution retrospective study of patients who underwent TMLI-based conditioning who subsequently developed EM relapse that was treated with radiation, and evaluated oncologic outcomes.

## Materials and methods

### Patient characteristics

Between 2006 and 2018, 254 patients with AML or ALL undergoing HCT with a TMLI-based conditioning regimen were enrolled in one of five prospective clinical trials. Following IRB approval, patients with a diagnosis of AML and ALL were included in this analysis. Of those patients, included patients were identified as having received a subsequent course of radiation treatment to extramedullary relapse. Patients without evidence of EM relapse or who did not receive RT treatment for EM relapse were excluded.

### Treatment

All patients underwent pre-transplant conditioning with high-dose chemotherapy and TMLI. Patients received one of four chemotherapy regimens on a per protocol basis, with regimens including busulfan/etoposide (VP-16), fludarabine/cyclophosphamide (CTX), fludarabine/melphalan, or VP-16/CTX (3, 4, 17–20). For all five trials, tacrolimus and sirolimus were administered for graft-versus-host disease (GVHD) prophylaxis. The institutional supportive care regimen was used to manage nausea, vomiting, mucositis, and infection risks. Patients were followed weekly with complete differential blood counts and comprehensive metabolic panel tests for the first 100 days after discharge. On days 30 and 100 after HCT, BM biopsy samples were obtained. Patients were then followed with annual BM biopsies at least two years post-transplant. EM relapses were found either on routine workup imaging or because of imaging confirmation of symptomatic lesions. EM relapses were confirmed based on either imaging and/or biopsy. EM recurrences were treated at the discretion of the hematologist and radiation oncologist using RT ± chemotherapy. Generally, radiatyion treatment volumes encompassed gross disease only. However, patients with CNS involvement received whole brain radiation, with or without craniospinal irradiation based on systemic therapy plan and cerebrospinal fluid cytology. Curative versus palliative intent was defined by the treating physicians.

### TMLI radiation therapy technique

**Figure 1:**
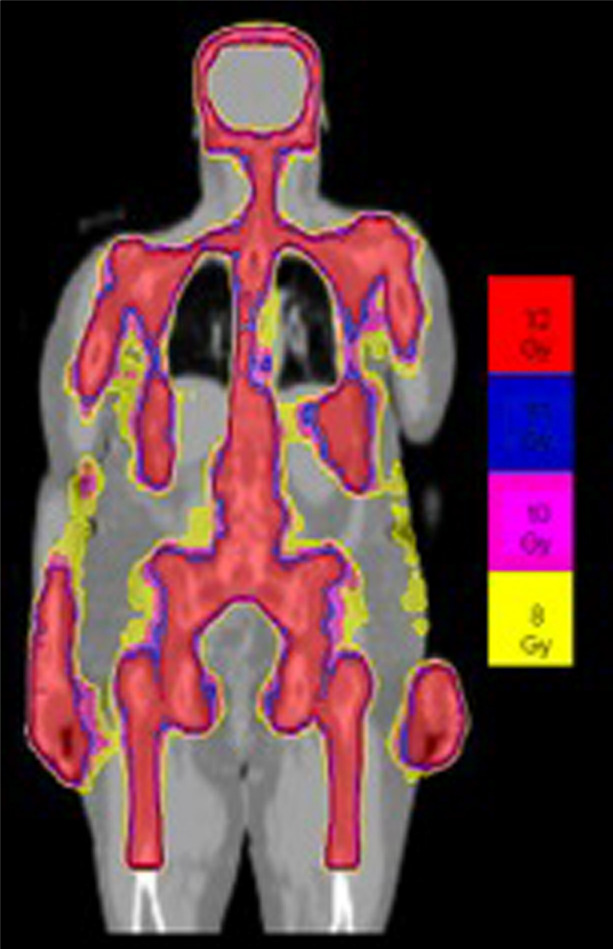
Color-coded TMLI plan shows dose to the targeted areas, with relative sparing of dose to critical organs.

Details of the TMLI radiation therapy technique have been published in previous studies (3, 21–23). All patients underwent scanning with a large-bore computed tomography simulator with 60-cm field of view (Phillips Medical System, Eindhoven, The Netherlands) for treatment planning purposes. Scans were obtained during shallow breathing, inspiration, and expiration to account for organ motion due to respiration. Patients were immobilized using a full-body Vac-lok bag (Civco Medical Systems, Kalona, IA) and a thermoplastic mask on the head and neck region. All patients were treated with a helical TomoTherapy unit (Accuray, Inc, Sunnyvale, CA), and the lower extremities were treated with a conventional linear accelerator through standard anteroposterior posteroanterior fields. The color-coded Tomotherapy TMLI dose distribution of a patient treated to 12 Gy is shown in **Figure 1**.

The patients were treated to a total dose of 12 Gy to 20 Gy using TMLI delivered twice daily in 1.25 Gy to 2 Gy fractions. The target structures were defined and included bone, major lymph node chains, testes, spleen, splenic-hilar lymph nodes, liver, portahepatic lymph nodes, and brain. In the dose escalation trials, only the bone, major lymph node chains, and testes (in some trials) were escalated for each dose level. All other targets remained at 12 Gy. Dose to organs at risk were optimally minimized and included orbit, lens, thyroid, oral cavity, mandible, parotids, larynx, hypopharynx, esophagus, lung, heart, breast, kidney, stomach, small and large intestines, rectum, and bladder. Target coverage was also optimized such that a minimum of 85% of target structure received the prescribed dose.

### Study definitions and statistical methods

Descriptive statistics were performed on the identified cohort of patients and the identified EM relapses. Patients and relapses were stratified by treatment intent (curative [radiation treating all known EM disease with or without systemic therapy] vs palliative [radiation limited to symptomatic lesions with or without systemic therapy]). Comparisons between continuous and categorical variables were made with Student’s t-test and chi-squared tests, respectively. Overall survival (OS) and progression-free survival (PFS) was estimated using the Kaplan-Meier method, as was local control (LC) of the treated relapses. These were defined from the date of the first fraction of radiation used for treatment of relapse following TMLI. Events for OS included death from any cause. Events for PFS included death or disease progression, whichever came first. Patients who did not experience an event at last follow-up were censored. For purposes of survival analyses, patients were stratified by treatment intent. All analyses were performed using open-source libraries in Python 3.8 (PSF, Wilmington, DE). Statistical significance was set at a p value of <0.05. Data were locked for analysis on January 31, 2021 (analytic date).

## Results

Patient characteristics are presented in **Table 1**. A total of 21 patients were identified who were subsequently treated with radiotherapy for EM relapse, with or without BM relapse, with patients having a median of 2 relapses treated (range: 1-16). At time of transplant, median age was 31 years (21-61 years). The median follow-up from date of transplant of these patients was 32.2 mo (3.9-154.1 mo). 13 (61.9%) of these patients were diagnosed with AML, accounting for 48 (71.6%) of the relapses, and 8 (38.1%) were diagnosed with ALL, accounting for 19 (28.4%) of the relapses. 6 patients had EM disease that had been treated prior to their transplant. 10 patients had received TMLI-based conditioning after induction failure, while 7 were treated after relapse and 4 were treated following complete response. Median time to initial relapse was 16.8 mo (0.9-51.5 mo). Twelve (57.1%) of patients did not have evidence of BM relapse prior to or at the time of initial EM relapse. Of the patients without evidence of BM disease at the time of initial relapse, only two subsequently developed BM relapse, although all but 4 received further systemic therapy after radiation and all but three had disease progression of extramedullary disease following radiation. Five (23.8%) patients had BM relapse prior to initial EM relapse while 4 (19.0%) presented with synchronous BM and EM relapse. Median time from transplant to relapse was 16.8 months.

**Table 1:** Patient characteristics.

Characteristic	All patients (N = 21)	Palliative (N = 10)	Curative (N = 11)	p
Age at transplantation (y) (median [range])	31.0 (21-61)	32.0 (25-61)	29.0 (21-57)	0.433
Follow-Up (m) (median [range])	38.8 (3.9-168.5)	20.9 (3.9-38.8)	61.0 (26.7-168.5)	<0.001
Time to Initial Relapse (m) (median [range])	16.8 (0.9-51.5)	7.2 (0.9-33.1)	18.1 (3.3-51.5)	0.099
Subsequent Courses of Radiation (median [range])	2.0 (1-16)	2.0 (1-16)	2.0 (1-10)	0.555
Race				0.483
Asian	1 (4.8%)	1 (10.0%)	0 (0.0%)	
Hispanic White	8 (38.1%)	3 (30.0%)	5 (45.5%)	
Non-Hispanic White	12 (57.1%)	6 (60.0%)	6 (54.5%)	
KPS				0.159
100	2 (9.5%)	0 (0.0%)	2 (18.2%)	
90	7 (33.3%)	2 (20.0%)	5 (45.5%)	
80	8 (38.1%)	6 (60.0%)	2 (18.2%)	
70	3 (14.3%)	1 (10.0%)	2 (18.2%)	
Unknown	1 (4.8%)	1 (10.0%)	0 (0.0%)	
HCTCI				0.418
0	13 (61.9%)	6 (60.0%)	7 (63.6%)	
1	1 (4.8%)	1 (10.0%)	0 (0.0%)	
2	2 (9.5%)	1 (10.0%)	1 (9.1%)	
3	1 (4.8%)	1 (10.0%)	0 (0.0%)	
4	2 (9.5%)	0 (0.0%)	2 (18.2%)	
5	1 (4.8%)	0 (0.0%)	1 (9.1%)	
Unknown	1 (4.8%)	1 (10.0%)	0 (0.0%)	
Diagnosis				0.038
ALL	8 (38.1%)	1 (10.0%)	7 (63.6%)	
AML	13 (61.9%)	9 (90.0%)	4 (36.4%)	
Disease Status at HSCT				0.472
1st CR	2 (9.5%)	1 (10.0%)	1 (9.1%)	
1st Relapse	5 (23.8%)	3 (30.0%)	2 (18.2%)	
2nd Relapse	1 (4.8%)	0 (0.0%)	1 (9.1%)	
3rd Relapse	1 (4.8%)	0 (0.0%)	1 (9.1%)	
>=3rd CR	2 (9.5%)	0 (0.0%)	2 (18.2%)	
Induction Failure	10 (47.6%)	6 (60.0%)	4 (36.4%)	
Prior EM Disease				0.73
No	15 (71.4%)	8 (80.0%)	7 (63.6%)	
Yes	6 (28.6%)	2 (20.0%)	4 (36.4%)	
Pretransplant Conditioning				0.338
Busulfan/VP-16	3 (14.3%)	2 (20.0%)	1 (9.1%)	
Fludarabine/CTX	3 (14.3%)	0 (0.0%)	3 (27.3%)	
Fludarabine/Melphalan	2 (9.5%)	1 (10.0%)	1 (9.1%)	
VP-16/CTX	13 (61.9%)	7 (70.0%)	6 (54.5%)	
TMLI Dose (cGy)				0.834
1200	8 (38.1%)	3 (30.0%)	5 (45.5%)	
1500	2 (9.5%)	1 (10.0%)	1 (9.1%)	
1600	2 (9.5%)	1 (10.0%)	1 (9.1%)	
1700	1 (4.8%)	1 (10.0%)	0 (0.0%)	
2000	8 (38.1%)	4 (40.0%)	4 (36.4%)	
First Relapse Site				0.213
BM	5 (23.8%)	4 (40.0%)	1 (9.1%)	
EM	12 (57.1%)	4 (40.0%)	8 (72.7%)	
EM & BM	4 (19.0%)	2 (20.0%)	2 (18.2%)	
Status				0.023
Deceased	15 (71.4%)	10 (100.0%)	5 (45.5%)	
Living	6 (28.6%)	0 (0.0%)	6 (54.5%)	

RT; radiotherapy, KPS; Karnofsky Performance Score, HCTCI; Hematopoietic Cell Transplantation-specific Comorbidity Index, ALL; acute lymphocytic leukemia, AML; acute myeloid leukemia, HSCT; hematopoietic stem cell transplant, CR; complete response, EM; extramedullary, VP-16; etoposide, CTX; cyclophosphamide, TMLI; total marrow and lymphoid irradiation, BM; bone marrow.

Descriptive statistics of the EM relapses are presented in **Table 2**. Radiation treatment intent for the first course of RT to EM relapses was curative in 11 (52.4%) patients and palliative in 10 (47.6%) patients. 67 relapse sites (median=2 sites/patient, range=1-16) were treated, with 16 (23.9%) treated with curative intent and 51 (76.1%) treated for palliation. The majority of EM relapses occurred in soft tissue (34), while there were 23 bone relapses, 6 nodal relapses, and 4 CNS relapses. 12 recurrences were treated with systemic therapy prior to RT. Furthermore, lesions treated with curative intent were more likely to have received initial treatment with systemic therapy (50% vs 17.6%, p=0.023).

**Table 2:** EM Relapse Characteristics.

Characteristic	All Sites (N = 67)	Palliative (N = 51)	Curative (N = 16)	p
RT Dose (cGy) (median [range])	24.0 (6.0-30.0)	22.0 (6.0-30.0)	24.0 (18.0-30.0)	0.006
Relapse Site				0.213
Bone	23 (34.3%)	19 (37.3%)	4 (25.0%)	
CNS	4 (6.0%)	2 (3.9%)	2 (12.5%)	
Lymph node	6 (9.0%)	6 (11.8%)	0 (0.0%)	
Soft tissue	34 (50.7%)	24 (47.1%)	10 (62.5%)	
Initial Treatment				0.023
Chemo to RT	17 (25.4%)	9 (17.6%)	8 (50.0%)	
RT	50 (74.6%)	42 (82.4%)	8 (50.0%)	
Durable Local Control				0.768
Yes	58 (86.6%)	45 (88.2%)	13 (81.2%)	
No	9 (13.4%)	6 (11.8%)	3 (18.8%)	

RT; radiotherapy, TMLI; total marrow and lymphoid irradiation;,BM; bone marrow, CNS; central nervous system, chemo; chemotherapy.

At the time of analysis, 6 patients were living, whereas 15 were deceased. One patient with CNS disease remains alive without evidence of disease at last follow-up. Among evaluable patients alive at the time of analysis, median follow-up was 38.8 months from the time of initiation of RT. Of the patients who were alive, 4 had initial EM relapse, 1 had concomitant EM and BM relapse, and 1 had BM relapse. 5 patients have no evidence of disease at last follow-up, of which 4 initially had EM relapse only (one went on to develop BM relapse salvaged by repeat transplant and chemotherapy) and 1 had BM relapse. Cause of death attributed to disease progression in all 15 patients.

**Figure 2:**
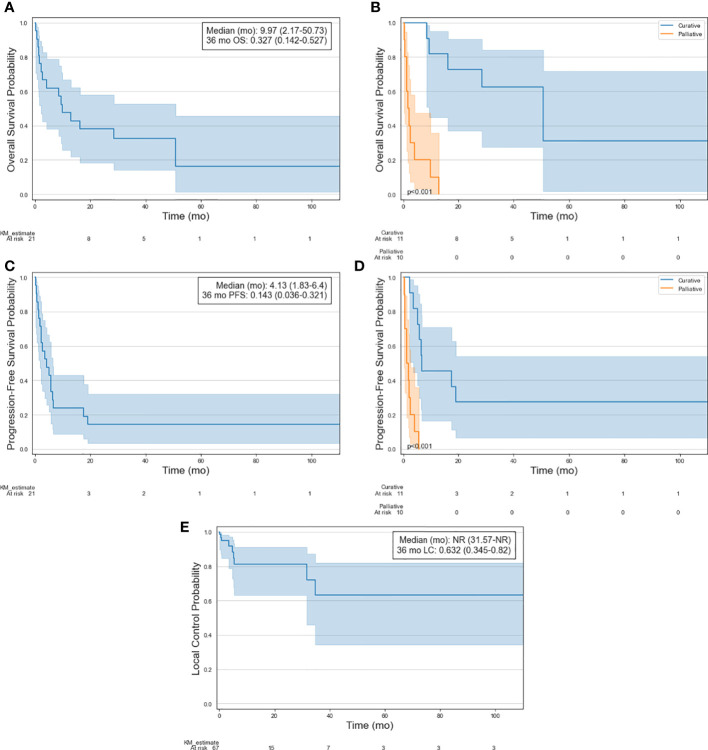
Kaplan-Meier curves of selected patient cohort illustrating **(A)** OS, **(B)** OS stratified by treatment intent, **(C)** PFS, **(D)** PFS stratified by treatment intent, and **(E)** LC of treated lesions.

Full survival curves for OS, PFS, and LC following RT treatment are visualized in **Figures 2A–E**. Following the initial course of subsequent RT, 1-year, 3-year and 5-year estimates of OS were 47.6%, 32.7% and 16.3%, respectively. Median OS was 10.0 months. OS was significantly better in patients treated with curative intent, with median OS of 50.7 months vs 1.6 months (p<0.001) and 5-year OS of 31.2% vs 0%. 1-year, 3-year and 5-year estimates of PFS were 23.8%, 14.3% and 14.3%, respectively. Median PFS was 4.1 months. PFS was significantly better in patients treated with curative intent, with median PFS of 6.6 months vs 1.3 months (p<0.001) and 5-year PFS of 27.3% vs 0%. Following RT, 86.6% of the sites had durable local control for the duration of follow-up. 1-year and 5-year estimates of LC were 81.2% and 63.2%, respectively. Of the 9 treated sites that progressed, 5 were initially treated with curative intent and four received a prescription dose of less than 20 Gy (range: 8-25 Gy). No secondary malignancies or significant radiation-induced toxicity were observed.

## Discussion

The incidence of EM relapse following TBI based conditioning regimens for HCT is estimated to be between 5% to 20% (5–8). An earlier study of patients treated with TMLI based conditioning regimens suggest similar outcomes, with the primary study evaluating patterns of failure demonstrating an EM relapse rate of 12.9% (9). Treatment of EM relapse in general is complicated, including the role of radiation (15, 16, 24). This question is relevant to patients treated with TMLI conditioning as well, based the difference in prior radiation distribution compared to TBI-based techniques leading to possible variations in the patterns and biology of EM relapse. This study demonstrated that not only is radiation an effective means of achieving local control in EM relapse following TMLI, select patients with more limited disease can achieve favorable long-term outcomes despite being high-risk even before their transplant and subsequent EM relapse.

In the present study, median time to relapse was 16.8 months, which is consistent with the prior study in TMLI (9). Common EM recurrence sites following TBI are the breasts, testes, and bone in ALL, and skin, the head and neck area, breast, bone and testes in AML (25). Of those sites, the testes, bone, and head and neck lymph nodes are included in the TMLI treatment volumes. Although bones represented a significant relapse site in the present study, there were few relapses in the other sites that are otherwise associated with relapse following TBI (3 breast recurrences [all AML], 1 scrotal recurrence [AML], and two head and neck nodal recurrences [both AML]).

When these EM recurrences did occur, RT was shown to be effective way of managing them, with 75% of relapses being treated with radiation first. This provided local tumor control for the duration of follow-up in 86.6% of sites, which is comparable to previously published series (26). Furthermore, in select cases RT proved to be an effective form of consolidative/salvage treatment, with 32.7% and 16.3% 3-year and 5-year OS following RT to first EM relapse, which is overall favorable given these were high-risk patients in the first place who had already relapsed following HCT. This appears to be largely driven by patients with limited relapse still amenable to salvage therapy, evidenced by significantly improved outcomes treated with curative intent due to have EM disease that could be contained in a radiation field. This is further evidenced by 80% of the long-term survivors initially presenting with a single EM relapse without BM involvement. These data are suggestive that in certain scenarios, radiation can be part of a curative regimen for patients with isolated EM relapse after TMLI and HCT. Beyond the efficacy of RT in treating EM relapse, the patients in this cohort did have overall favorable survival outcomes compared to historical controls.

It is important to note that the majority of the patients treated on the included trials were higher risk with worse prognosis compared to most patients undergoing traditional TBI. This is evidenced in this study’s cohort, where only 4 patients (19.0%) were in complete remission prior to transplantation. In a study of patients not in CR treated with HCT after relapse or induction failure, Duval et al. reported 3-year overall survival rates of 19% for AML and 16% in younger patients (27). On subset analysis, 3-year OS was 42% and 46% in AML and ALL, respectively, for patients even with the best prognostic factors. In another study, Ganzel et al. reported median OS of 6 months and 5 year OS of 10% following AML relapse (28). In a retrospective study of patients with relapsed/refractory AML, Brandwein et al. sought to examine outcomes following intensive therapy (23% of patients), non-intensive therapy (33%), and best supportive care (44%) (29). Intensive therapy was defined as re-induction with a different intensive induction regimen following induction failure with 2 other regimens. Non-intensive therapy was defined as a hypomethylating agent (HMA) or low-dose cytarabine, with or without another chemotherapy agent. Patients who could not receive either intensive therapy or non-intensive therapy would receive best supportive care. The 5-year OS rates of the entire cohort was 12.6% and was 36.7%, 7.0% and 4.0% for the intensive therapy, non-intensive therapy, and best supportive care groups, respectively. Our relapsed TMLI cohort presented here is comparable to the intensive therapy described by Brandwein et al, particularly in patients amenable to curative intent treatment (29). The median OS outcomes were 13.6, 9.4, and 2.0 months for the intensive therapy, non-intensive therapy, and best supportive care groups, respectively, compared to 10.0 months in our overall cohort and 51 months in our curative intent cohort.

These outcomes suggest that there might be a role for radiation in managing EM relapse following TMLI, given they may be chemo-resistant after extensive pretreatment with systemic therapies. Indeed, in one retrospective study of relapsed leukemia treated with high-dose chemotherapy followed by donor leukocyte infusion, Choi et al. reported that all patients who relapsed following initial complete response relapsed at extramedullary sites (24). In another study, Ginsberg et al. report on treatment of isolated extramedullary relapse in children of AML (15). Of the 6 patients still alive at last follow up, all received local radiation to the EM relapse plus or minus TBI and subsequent transplant. Overall, these studies and our study support current guidelines from the International Lymphoma Radiation Oncology Group (ILROG), where radiation is recommended for patients with isolated chloroma and inadequate response to chemotherapy, with isolated recurrence after HCT (16). Nevertheless, despite excellent local effect, there is no doubt that EM disease often occurs alongside BM disease or is a harbinger of subsequent BM disease (30, 31). However, it is possible that this pattern could be mitigated by the dose escalation to the bone marrow accomplished by TMLI, evidenced by the fact that only 2 of the 12 patients presenting with isolated EM relapse included in our cohort went on to develop BM relapse. This lends further support to aggressive local treatment of limited EM relapse following TMLI. Our data are also consistent with ILROG guidelines for more advanced disease, with goal being palliation of symptomatic lesions, due to high rates of local control.

This study has several limitations. This is a heterogeneous cohort, including a wide range in dosimetry, GVHD prophylaxis, chemotherapy, and donors, that is not intended to fully represent the patterns of failure of TMLI. For example, it is possible that different systemic therapy regimens may have differential effects on the occurrence of EM relapse, underlying biology, and treatment response. Our cohort is certainly not inclusive of all EM relapses and therefore cannot estimate incidence of relapse beyond a rough estimate, which further applies to the dosimetry and outcomes of relapse following TMLI. Specifically, with regards to outcomes, it is possible this cohort represents a more favorable cohort of patients, given that 57.1% presented with initial EM relapse, as compared to approximately 24.3% in the largest published TMLI cohort (9). Initial EM relapse has been shown to have better prognosis that initial BM ± EM relapse (32). This imbalance is potentially related to patients with more limited systemic disease being selected for subsequent RT but suggests additional roles for RT in limited leukemic disease. Additionally, though our study shows favorable outcomes for EM relapse following TMLI treated with RT, our cohort does not include chemotherapy alone as a standard comparator arm. Therefore, it is not possible to compare outcomes to patients who did not receive any radiation, although half the patients in the curative cohort did receive chemotherapy before radiation with insufficient response, which does lend credence to radiation being a valuable option for resistant disease per ILROG guidelines (16). These limitations will best be addressed by future retrospective studies specifically evaluating patterns of failure in patients treated with TMLI, as well as further planned prospective studies on the role of TMLI in high-risk leukemia. Despite these limitations, all patterns of relapse, outcomes, and dosimetry are from this study are comparable to the largest published series investigating EM relapse following TMLI (9), and therefore support continued investigation of TMLI and the role of RT as a subsequent salvage therapy.

## Conclusions

To our knowledge, this is the first analysis to date of salvage/palliative radiation treatment for patients treated with TMLI and HCT who developed EM relapses. RT is an effective modality to treat EM relapse in patients with acute leukemia who were previously treated with TMLI, offering a high probability of durable local control. Furthermore, these patients did not have significantly different OS compared to historical controls, with patients treated with curative intent demonstrating favorable outcomes. These data suggest a subset of patients with limited disease at relapse can potentially be salvaged with the help of radiation. Further investigation of the treatment of EM relapse following TMLI (or HCT in general), in addition to overall patterns of failure, outcomes, and toxicity is warranted.

## Data availability statement

The raw data supporting the conclusions of this article will be made available by the authors without undue reservation.

## Ethics statement

The studies involving human participants were reviewed and approved by City of Hope National Medical Center Institutional Review Board. Written informed consent for participation was not required for this study in accordance with the national legislation and the institutional requirements.

## Author contributions

The authors confirm contribution to the paper as follows:

Study conception and design: CL, SD. Data collection: CL. DY. Analysis and interpretation of results: CL, DY. Draft manuscript preparation: CL, SD. All authors contributed to the article and approved the submitted version.

## Conflict of interest

SD has received grant funding from Bayer.

The remaining authors declare that the research was conducted in the absence of any commercial or financial relationships that could be construed as a potential conflict of interest.

## Publisher’s note

All claims expressed in this article are solely those of the authors and do not necessarily represent those of their affiliated organizations, or those of the publisher, the editors and the reviewers. Any product that may be evaluated in this article, or claim that may be made by its manufacturer, is not guaranteed or endorsed by the publisher.
